# Pararectal hernia: literature review and surgical repair techniques in the era of robotic surgery

**DOI:** 10.1093/jscr/rjab378

**Published:** 2021-08-31

**Authors:** Rebecca Glanzer, Breanna O’Neil, Hassan Turaihi

**Affiliations:** Sanford School of Medicine, Vermillion, SD, USA; Department of General Surgery, Sanford School of Medicine, Sioux Falls, SD, USA; Department of General Surgery, Sanford Health, Sioux Falls, SD, USA

**Keywords:** pararectal hernia, robotic repair, mesh

## Abstract

A pelvic hernia is the protrusion of intraperitoneal or extraperitoneal contents into the perineum through a defect in the pelvic floor. Pelvic hernias are rare with no gold standard method for repair. After abdominoperineal resection, a commonly cited incidence of perineal hernia is 1%. Here, we describe a robotic repair of a pararectal hernia in a post-menopausal women presenting with rectal herniation through a pelvic floor defect causing issues with fecal urgency and incontinence.

## INTRODUCTION

Perineal hernias are the protrusion of intraperitoneal or extraperitoneal contents into the perineum through a defect in the pelvic floor. Perineal hernias can be classified as anterior or posterior perineal hernias based on their location relative to the transverse perineal muscles. A primarily acquired perineal hernia is most often seen in older, multiparous women. Secondarily acquired perineal hernias are incisional hernias associated with extensive pelvic operations, the most common of which is abdominoperineal resection (APR). After APR, a commonly cited incidence of perineal hernia is 1%, although some sources report incidence anywhere from 0.8 to 12% over long-term follow-up [1, 2].

## CASE REPORT

**Figure 1:**
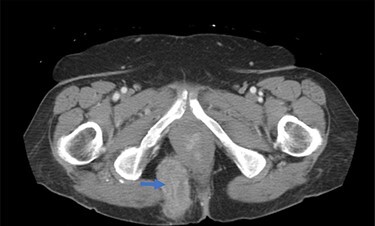
Preoperative CT demonstrating pararectal hernia (blue arrow).

A 62-year-old post-menopausal female presented with worsening fecal urgency, incontinence and a corresponding perianal bulge that was increasing in size over the past 2 years. The patient had a recent history of significant weight loss through diet modification. Medical history was significant for two previous vaginal deliveries, 15-pack per year smoking history, hypothyroidism, hiatal hernia, previous CVA and chronic obstructive pulmonary disease. Fifteen years prior, the patient underwent a total vaginal hysterectomy, including a McCall culdoplasty, anterior and posterior repairs, sacrospinous ligament suspension and tension-free vaginal taping to treat symptomatic cystocele, rectocele and descent of the uterus. A previous computed tomography (CT) demonstrated a heterogeneously enhancing mass extending from the right posterolateral margin of the lower vaginal cuff and perirectal region into the ischioanal fossa, suggestive of, but not definite for a soft tissue mass (Fig. 1). Upon clinical exam, the patient was noted to have a defect in the right levator muscles complex with complete herniation of her rectum through this defect. This hernia created pocking of the stool and difficulties with stool emptying. In addition, the patient had to adjust her sitting position to avoid unwanted stool leakage as a result of spontaneous hernia reduction. The patient was offered an abdominoperineal resection; however, discussion of laparoscopic interventions with the DaVinci robot quickly became the best option for repair. Informed consent was obtained for robotic repair, and routine preoperative colonoscopy was performed.

**Figure 2:**
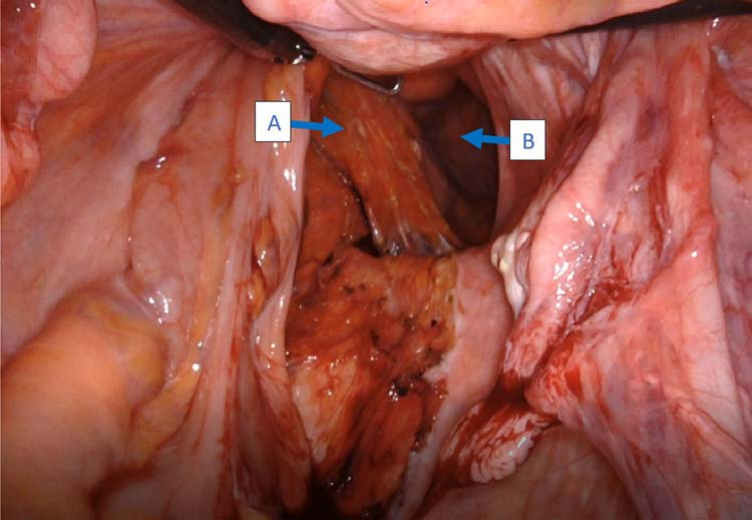
Intraoperative view of the rectum (**A**) herniating through a defect in the pelvic wall (**B**).

Intraoperatively, cystoscopy and intraurethral ICG injection were performed as a routine practice to aid with the identification and protection of ureters in redo pelvic surgeries. The abdomen was accessed using the Veress needle technique, and robotic trocars were placed in transverse fashion at the level of the umbilicus. Initially, a medial-to-lateral mobilization of the descending colon was performed. The mesorectal fascial plan was entered posteriorly and the rectum was circumferentially mobilized. The pelvic defect was encountered on the right side, and it was noted that the rectum was redundant, folded on itself and contained by the hernia sac (Fig. 2). Using meticulous dissection, it was reduced completely. The hernia roof consisted of the gluteal skin only. The hernia neck was wide and consisted of defects in the levator muscle. The anal canal formed part of the hernia wall.

**Figure 3:**
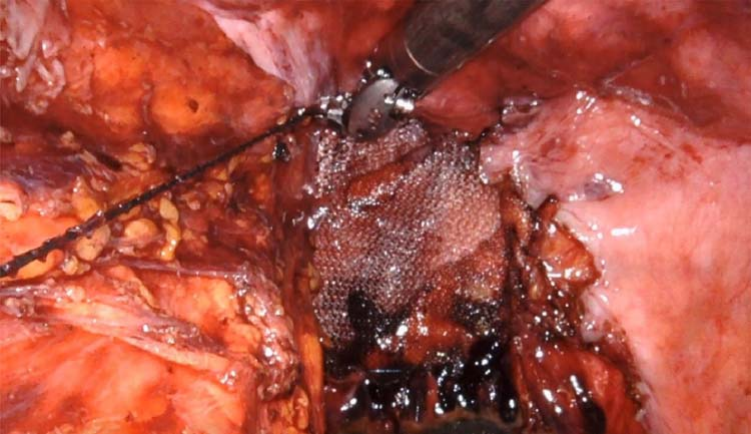
Intraoperative repair with 8 × 8 cm Phasix mesh.

**Figure 4:**
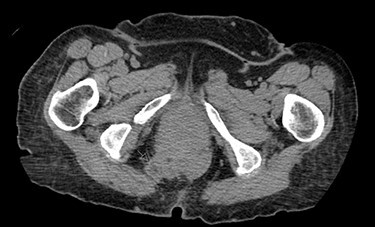
Post-operative CT demonstrating mild fluid in pelvis and resolution of the hernia (black arrow).

Once the hernia was completely reduced, the hernia sac was resected. The muscular defect of the pelvic floor was delineated. The pelvic floor was reconstructed, and the muscular defect was reapproximated from lateral to medial using 3-0 V-Loc running sutures in a tension-free manner. An 8 × 8 cm synthetic bioabsorbable Phasix mesh was used to reinforce the repair (Fig. 3). To prevent the rectum from falling back into the pelvis, a suture rectopexy was performed in the traditional fashion by clearing the sacral promontory from overlying soft tissue, exposing the periosteum of the sacral promontory and using silk sutures to attach the rectum to the sacrum. The pelvis was reperitonealized using running 3-0 V Loc absorbable sutures. Post-operatively, the patient had return of bowel function, adequate pain control, tolerated a soft diet and was discharged after 2 days. The patient had significant improvement in her fecal incontinence at her 4-week follow up visit, and post-operative CT demonstrated resolution of the hernia (Fig. 4).

## DISCUSSION

Due to the low incidence and a variety of options for repair, there is a lack of consensus on the superior surgical approach for repair of pararectal hernias. The surgical principals for all methods of repair remain the same: mobilization and reduction of the contents and closure of the defect under no tension. Additionally, direct suture placement, mesh placement or autogenous tissues, such as muscle flaps, can be used to reinforce the repair [4]. A perineal approach has been associated with a lower success rate due to the difficulty with direct visualization, which leads to a higher risk of injury to pelvic structures as well as difficulty in mobilizing adherent bowel and securing mesh. An open transabdominal repair allows mobilization of herniating small bowel under direct vision and the ability to take down intra-abdominal adhesions that limit the maneuverability in other approaches. A newer laparoscopic transabdominal approach offers the same advantages of open transabdominal repair with fewer reported complications and less-invasive repairs when compared with other approaches [3, 5, 6]. A combined laparoscopic-perineal approach allows for excellent visualization, safe mobilization and easy reduction of herniating viscera, although the magnitude of this procedure is associated with higher morbidity [1, 5, 7]. In a series of 21 pelvic hernia repairs from the past two decades, a low rate of recurrence was seen across all repair methods [1]. Interestingly, one patient in this series had a reported history of hysterectomy with rectocele repair similar to our patient, which may suggest this as a potential index operation. Ultimately, the decision to use a particular approach is based on a multitude of patient-specific factors for which no ‘gold standard’ exists.

In this case report, we present a robotic-assisted abdominal repair of a pararectal hernia with the use of 8 × 8 cm Phasix mesh for reinforcement. The robotic approach provided better visualization and easier manipulation within the deep pelvis than would have been achieved with other approaches while maintaining the benefits of a minimally invasive repair as demonstrated in other similar reports [2, 8]. The DaVinci robot allowed for clear visualization of the herniation involving the levator muscle that protruded through a thin gluteal muscle and allowed for the closure of a large defect with minimal tension.

## CONFLICT OF INTEREST STATEMENT

None declared.
